# Genome Insights and Identification of Sex Determination Region and Sex Markers in *Argyrosomus japonicus*

**DOI:** 10.3390/genes15121493

**Published:** 2024-11-21

**Authors:** Yike Liu, Wanbo Li, Dinaer Yekefenhazi, Xianfeng Yang, Qihui Zhu, Kun Ye, Fang Han, Dongdong Xu

**Affiliations:** 1 Key Laboratory of Healthy Mariculture for the East China Sea, Ministry of Agriculture and Rural Affairs, Jimei University, Xiamen 361021, China; 13207030273@163.com (Y.L.); li.wanbo@jmu.edu.cn (W.L.); s2745005@ed.ac.uk (D.Y.); kye1015@jmu.edu.cn (K.Y.); 2 Agro-Tech Extension Center of Guangdong Province, Guangzhou 510145, China; xianfengyang76@163.com; 3 Key Lab of Mariculture and Enhancement of Zhejiang Province, Zhejiang Marine Fisheries Research Institute, Zhoushan 316100, China; zqhpp@sina.com

**Keywords:** *Argyrosomus japonicus*, genome assembly, sex determination, sex markers

## Abstract

Background: *Argyrosomus japonicus*, a member of the Sciaenidae family, is widely distributed across the sea areas near China, Japan, Australia, and South Africa. The aim of this study is to provide a high-quality genome with new technology and to understand the sex determination mechanism of this species. Methods: We generated a high-quality chromosome-level genome for *Argyrosomus japonicus* using PacBio HiFi and Hi-C sequencing technologies. To map the sex determination region, we employed re-sequencing data from 38 *A. japonicus* and conducted genome-wide association studies (GWASs) on sex phenotypes. Results: Utilizing Hifiasm, we assembled a 708.8 Mb genome with a contig N50 length of 30 Mb. Based on Hi-C data, these contigs were organized into 24 chromosomes. The completeness of the assembly was assessed to be 99% using BUSCO, and over 98% according to Merqury. We identified a total of 174.57 Mb of repetitive elements and annotated 24,726 protein-coding genes in the genome. We mapped a 2.8 Mb sex determination region on chromosome 9, within which we found two sex-linked markers. Furthermore, we confirmed that the XX-XY sex determination system is adopted in *A. japonicus*. Conclusions: The findings of this study provide significant insights into genetic breeding, genome evolution research, and sex control breeding in *A. japonicus*.

## 1. Introduction

*Argyrosomus japonicus* belongs to the order Perciformes and the family Sciaenidae, which includes fish of high economic and nutritional value. Globally, there are approximately 70 genera and 270 species within this family, with 35 species and 18 genera found along the coast of China [1]. Sciaenidae species constitute a significant portion of the marine aquaculture industry in China, and *A. japonicus* plays a vital role in marine aquaculture in southern China. This species is commonly found in tropical waters, with a worldwide distribution that includes the East China Sea, the South Japan Sea, and coastal areas near Australia and South Africa [2,3]. *A. japonicus* is a predatory fish that inhabits estuaries and nearshore regions, reaching approximately 850 mm at 50% maturity for females and 778 mm for males [3,4].

Due to marine environment degradation and overfishing, the wild population of *A. japonicus* has significantly declined [5,6]. In 2018, it was classified as endangered on the IUCN Red List of Threatened Species [5]. This species is valued for meat and fish maw production in East Asia, prompting a rise in aquaculture practices in China to offset the decline in wild populations. It can be cultured in both marine net cages and brackish water ponds [7], facilitating the expansion of aquaculture and increasing efficiency.

Currently, studies on *A. japonicus* are relatively scarce. Research has investigated the species’ temperature preferences [8] and the effects of varying light intensities on feed conversion efficiency and growth [9]. Different feeding methods have also been explored to enhance our understanding of growth patterns and changes in body size [10]. Recently, a high-density genetic linkage map spanning 2550 cM was constructed using 2b-RAD sequencing, covering 98.61% of the genome. QTL analysis based on this map identified 25 QTLs associated with growth traits such as weight and length. Additionally, the sex-specific maps constructed by the study revealed a higher recombination rate in male than in female, with a female–male ratio of 1:1.44, which might suggest female heterogamy (ZZ/ZW system) in *A. japonicus* [11].

Research has also focused on habitat range, life history, and mortality rates in wild populations [3,12]. For example, Silberschneider et al. conducted a comprehensive study of the *A. japonicus* population in southeastern Australia, examining age, growth patterns, sexual maturity, and the impacts of overfishing [13]. In 2009, a study isolated 15 polymorphic microsatellite DNA loci from *A. japonicus* in southern Australia, and assessing genetic variation and population structure across the species’ natural distribution [14]. Another study revealed signs of genetic bottlenecking along the South African coast, indicating a decline in effective population size due to ongoing overfishing pressures, despite existing management measures that are undermined by poor enforcement [6]. Collectively, these studies highlight the decline of wild populations of *A. japonicus* and underscore the need for effective conservation strategies and management practices.

Research indicates that males have a shorter maturation period compared to females in *A. japonicus*, with females maturing at around 4–5 years and reaching a body length of 68 cm, while males mature at approximately 2–3 years and reach 51 cm. This difference suggests the potential benefits of all-female aquaculture [15]. Therefore, genetic markers are essential in the aquaculture of this species. Also, the sex system (XX/XY or ZZ/ZW) can be determined through genotypes of accurate sex markers. Previously, an assembly of *A. japonicus* (sampled in China, CN) of 673.7 Mb, with a contig N50 length of 18.4 Mb, has been released [16]. In addition, a draft genome of *A. japonicus* sampled in South Africa (SA) has recently been reported, with a size of 742 Mb [17]. In this study, we generated a high-quality genome assembly with new technology, mapped the sex determination region with resequenced samples, and obtained reliable genetic markers for sex identification. These results will enhance our understanding of the biological aspects of *A. japonicus*, including gene structure, evolution, germplasm resource preservation, and selective breeding strategies in aquaculture.

## 2. Materials and Methods

### 2.1. Sample Collection

A total of 65 *A. japonicus* (30 males and 35 females) were collected from two aquaculture farms in Zhangzhou, Fujian province, China. Fin samples from each fish were preserved in 95% alcohol and stored in a −20 °C freezer. Muscle, brain, heart, liver, spleen, gills, intestine, and gonad tissues from one male and one female were collected and stored in liquid nitrogen for RNA sequencing, PacBio, and Hi-C sequencing. The sex of the sampled individuals was determined by inspecting the dissected gonads, with verification through histological sectioning and hematoxylin–eosin staining.

### 2.2. Species Identification of A. japonicus

To identify the species of the individuals we collected, we designed a primer pair (forward primer: 5′-ACAGCCTTGAGCCTCCTAAT-3′ and reverse primer: 5′-TGTTGGTAAAGAATTGGGTC-3′) to amplify the COI gene of the 65 samples. We also downloaded all available COI sequences for *A. japonicus* from the NCBI database (https://www.ncbi.nlm.nih.gov/nuccore, accessed on 6 May 2023). The PCR amplification conditions were as follows: initial denaturation at 95 °C for 5 min, followed by 35 cycles of denaturation at 95 °C for 30 s, annealing at 52.5 °C for 30 s, extension at 72 °C for 1 min, and a final extension at 72 °C for 5 min. The PCR products were visualized using electrophoresis on a 1.5% agarose gel and subsequently sent for Sanger sequencing at Xiamen Borui Biotechnology Co., Ltd. (Xiamen, China).

After merging all obtained sequences, a multiple sequence alignment was performed using MUSCLE in MEGA11 [18]. Phylogenetic analysis was conducted using the Neighbor-Joining method with a bootstrap value of 1000. After testing 5 models in MEGA11, the Kimura 2-Parameter (K2P) model (with highest maximum likelihood) was used to construct the NJ tree, while genetic distances were calculated with the P-distance model.

### 2.3. Genome Sequencing, RNA Sequencing and Hi-C

DNA was extracted from fins using the phenol/chloroform method. The qualified DNA were fragmented using the Covaris M220 ultrasonic disruptor, and purified with Agencourt AMPure XP magnetic beads (Beckman Coulter, Mumbai, India). In the library preparation, the fragmented DNA was end-repaired and A-tailed, and Illumina adapters were then ligated. The library was then purified and amplified with PCR, and sequenced on the Illumina NovaSeq X platform, with paired-end reads of 2 × 150 bp. High-molecular-weight DNA was also extracted from the muscle of a male using the phenol/chloroform method. The DNA was then sheared to 15–20 kb using a g-TUBE (Covaris, Woburn, MA, USA). The library construction process included sample preparation, DNA fragmentation, the repair of DNA damage and ends, the annealing of sequencing primers to SMRTbell templates, and long-read sequencing on the PacBio Sequel IIe platform.

Total RNA was extracted from frozen tissues, including muscle, brain, heart, liver, spleen, gills, intestine, and gonads, using TRIzol reagent (Invitrogen, Waltham, MA, USA) according to the manufacturer’s instructions. The extracted RNA was pooled in equal amounts and sequenced on the Illumina NovaSeq X platform, with paired-end reads of 150 bp.

High-throughput chromosome conformation capture (Hi-C) libraries were constructed using muscle tissue. Briefly, the muscle tissue of the male was ground in liquid nitrogen and cross-linked with formaldehyde. To quench the cross-linking reaction, 2.5 M glycine was added. Samples were collected by centrifugation and resuspended in lysis buffer. After lysis, the nuclei were resuspended and digested overnight with the 4-cutter restriction enzyme MboI (400 units). DNA ends were then labeled with biotin-14-dCTP, and the cross-linked fragments underwent blunt-end ligation. The nuclear complexes were reverse cross-linked by incubating with proteinase K at 65 °C. DNA purification was performed using phenol/chloroform extraction, and non-ligated fragment ends were treated with T4 DNA polymerase to remove biotin. Finally, Illumina paired-end sequencing adapters were ligated onto the A-tailed DNA fragments, and the Hi-C sequencing libraries were sequenced on the Illumina NovaSeq X system.

### 2.4. Genome Assembly and Evaluation

Jellyfish (v2.3.0) was used to count the k-mers from the high-coverage resequencing data [19], and genome size was estimated using various k-mer parameters: 19, 23, 27, and 31. Genomescope (version 2.0) generated frequency distribution plots, calculated heterozygosity rates, assessed the proportion of repetitive sequences, and analyzed GC content [20].

Hifiasm (v0.13.0-R307) was utilized to assemble the HiFi reads into contigs [21]. First, sequencing errors were corrected through sequence alignment. A phased string graph was then generated based on the overlaps among the sequences. Hifiasm randomly selected one side of the bubble to construct the primary assembly, while the other side became the alternate assembly. Juicer (v1.6) processed the Hi-C dataset [22], and 3D-DNA (v180419) was employed for chromosome-level scaffolding based on the Hi-C reads [23]. Additionally, manual corrections to the scaffolded assembly were made using Juicerbox (v1.11.08) [24]. The quality and completeness of the assembly were assessed using Benchmarking Universal Single-Copy Orthologues (BUSCO) (v5.1.2) and Merqury software (v1.3) [25,26].

### 2.5. Annotation of Repeat Elements and Gene Annotation

RepeatModeler (v1.0.9) was employed to construct a species-specific repeat sequence database through de novo prediction [27]. Subsequently, RepeatMasker (v4.0.6) was used to detect repetitive sequences and annotate repeat elements in the assembled genome [28], utilizing both the Repbase library and the locally constructed library from RepeatModeler. All identified repeats were then merged.

Gene annotation for *A. japonicus* was performed using three approaches: de novo prediction, transcriptome prediction, and protein homology-based gene prediction. Trinity (v2.4.0) was used to assemble the cleaned RNA-seq reads [29]. PASA (v2.1.0) optimized the results from Trinity and extracted open reading frames (ORFs) for training Augustus (v3.2.3) [30,31], which involved an iterative process to identify optimal parameters. For protein homology-based gene prediction, Tblastn aligned the *A. japonicus* genome with the protein sequences of nine other species, namely, *Cynoglossus semilaevis*, *Danio rerio*, *Takifugu rubripes*, *Dicentrarchus labrax*, *Gasterosteus aculeatus*, *Larimichthys crocea*, *Lates calcarifer*, *Oreochromis niloticus*, and *Oryzias latipes*. Cufflinks assembled transcripts after aligning the transcriptome sequences to the genome using Tophat (v2.1.1) [32]. Finally, all sequences from these gene prediction models were merged using Evidence Modeler (EVM) (v1.1.1) [33]. All predicted genes were compared to the NCBI non-redundant protein (Nr) database for functional annotation.

### 2.6. Mapping Sex Determination Region and Development of Sex Markers

The sequencing reads were aligned to the genome of *A. japonicus* using BWA (v0.7.17-r1188) [34], and SNP calling and filtering were performed using GATK (v4.2.0.0) [35]. The filtering parameters for GATK included QD > 2.0, MQ > 40.0, FS < 60.0, ReadPosRankSum > −8.0, and MQRankSum > −12.5. Genome-wide association analysis was conducted to map the sex determination region. We used PLINK (v1.90) to analyze the association between the filtered SNPs and the sex phenotype without any covariates [36].

We screened for insertions and deletions (indels) near the highest association SNPs as candidate sex markers. PCR primers for these indels were designed using Primer Premier 5.0. The amplification conditions were as follows: initial denaturation at 95 °C for 5 min, followed by 30 cycles of denaturation at 95 °C for 30 s, annealing at 60 °C for 30 s, extension at 72 °C for 30 s, and a final extension at 72 °C for 5 min. A 3% agarose gel was used for electrophoresis to separate the PCR products.

## 3. Results

### 3.1. Species Identification Results of A. japonicus

Using the designed primers, we amplified 608 bp sequences of the COI gene from the sampled individuals. All amplified COI sequences were utilized to construct a phylogenetic tree based on a consensus sequence of 570 bp, along with COI sequences from *Argyrosomus hololepidotus*, *Argyrosomus inodorus*, *A. japonicus*, *Collichthys lucidus*, *Larimichthys crocea*, *Nibea coibor*, and *Micropterus salmoides*, which were downloaded from GenBank (https://www.ncbi.nlm.nih.gov/genbank/, accessed on 6 May 2023) (Figure S1). Our collected samples clustered closely with previously published COI sequences of *A. japonicus*, with genetic distances generally less than 0.02. Furthermore, the genetic distance between species was over ten times greater than the distance within species (Figure S2), which aligns with the findings for species identification using COI gene sequences [37].

### 3.2. Results of Genome Assembly, Quality Evaluation and Functional Annotations

The genome size of *A. japonicus* was estimated to be between 702.9 Mb and 755.6 Mb using the K-mer method (Table 1, Figure S3). HiFi reads were first assembled with Hifiasm, resulting in a genome size of 708.8 Mb composed of 181 contigs. The N50 length was 30 Mb, and the N90 length was 22.54 Mb. Hi-C data were used to merge the scaffolds into 24 chromosomes, yielding a final chromosome-level genome of 708.02 Mb, with 98% of the contigs mapped onto the chromosomes (Table 2).

The quality of the genome was assessed using BUSCO and Merqury. BUSCO analysis revealed that 99% of the 3640 genes in the actinopterygii_ODb10 database were complete in our assembly; specifically, 3602 genes were identified as complete, with 3571 as single-copy genes and 31 as multi-copy genes. In the Merqury assessment, the genome integrity, measured by k-mer counts, showed that the completeness of our assembly was 98.2% based on HiFi reads and 98.3% based on Illumina reads. Additionally, the quality values (QV) estimated by Merqury were 65.8 for HiFi reads and 51.2 for Illumina reads.

We identified 172.57 Mb of repetitive sequences, accounting for 24.64% of the *A japonicus* genome. These repetitive sequences comprised 3.43% DNA repeat elements, 3.26% simple repeats, 2.75% long interspersed nuclear elements (LINEs), 1% long terminal repeats (LTRs), and 0.6% short interspersed nuclear elements (SINEs) (Table 3). A total of 24,726 genes were annotated in the assembly.

### 3.3. Mapping the Sex Determination Region and Possible Candidate Genes

**Figure 1:**
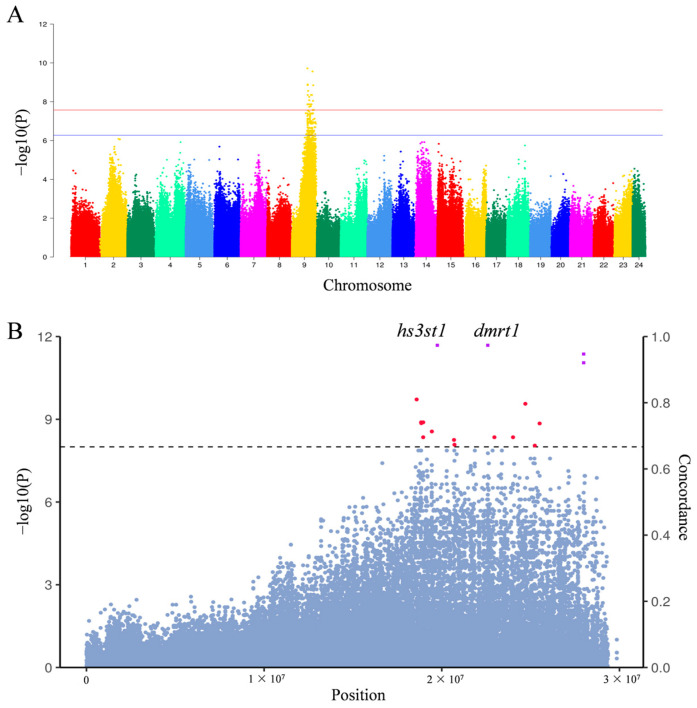
Location of sex determination region in *A. japonicus*. (**A**) Manhattan plot of *A. japonicus* association analysis. (**B**) Manhattan plot of chromosome 9 association analysis and scatter plot with a coincidence of more than 90%. The blue dots indicate all SNPs on chromosome 9, the red dots indicate SNPs on chromosome 9 that exceed the threshold line, and the purple box indicates SNPs with a compliance greater than 90%. If a purple square resides in an annotated gene, the gene name is labeled.

To map the sex determination region of *A. japonicus*, we sequenced 38 individuals (18 males and 20 females) using the Illumina NovaSeq X platform. The samples generated approximately 234.55 Gb of sequencing data, with an average depth of around 8.7×. BWA was employed to align the sequencing reads to our assembly, while GATK was used for SNP calling and hard filtering. In total, we obtained a final set of 1,878,032 high-quality SNPs. The genome-wide association analysis (GWAS) of the sex phenotype revealed the highest significance on chromosome 9, spanning an approximate interval of 3 Mb. Additionally, we identified SNPs where genotype and sex phenotype matched with an XX-XY sex determination system (i.e., heterozygotes for males and homozygotes for females). Notably, all SNPs with over 90% consistency were located between 19.74 Mb and 22.60 Mb on chromosome 9, overlapping with the GWAS mapped region (Figure 1). This region includes a total of 96 genes, among which *hs3st1*, *pkn2*, and *snx2* show high expression in ovaries, while *slc2a8*, *odf2*, *cfap157*, *ttc16*, and *tex2* show high expression in testes in humans. The top ten significantly associated genes are listed in Table S1. Notably, the *dmrt1* gene is located in the center of this region.

### 3.4. Development of Sex Markers

**Figure 2:**
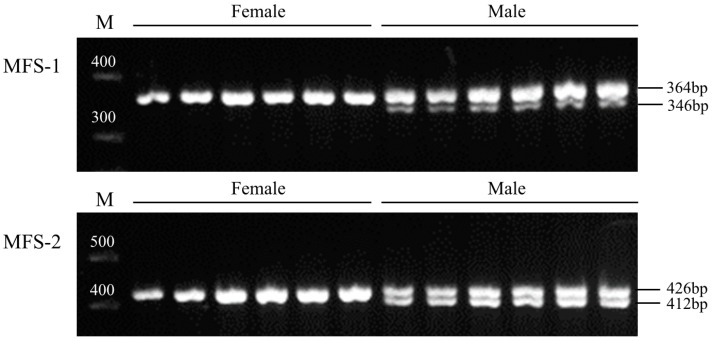
Verification of two molecular markers in random samples. (MFS-1) Verification of 18 bp deletion. (MFS-2) Verification of 14 bp deletion.

We screened for insertions and deletions (indels) in the regions where the top associated SNPs were located and identified two indels, measuring 18 bp and 14 bp in length. Both indels were found in the intronic region of the *dmrt1* gene. We validated these indels in 38 sequenced samples, along with an additional 27 samples. The 18 bp indel is situated in the third intron of *dmrt1*, resulting in PCR product sizes of 364 bp for males and 346 bp for females (Table 4, Figure 2). The 14 bp indel is located in the fifth intron of *dmrt1*, with product sizes of 426 bp for males and 412 bp for females (Table 4, Figure 2). The genotypes of these two markers corresponded accurately with the sex of the samples, achieving an accuracy rate of 97.30% and 100% across the two sample batches.

## 4. Discussion

Fish play a significant role in the animal kingdom, with numerous species accounting for over half of all known vertebrates [38]. Research into fish sex determination has gained increasing interest, particularly as many fish species exhibit sexual dimorphism while lacking heteromorphic sex chromosomes. Understanding the mechanisms of sex determination and other genetic factors is greatly facilitated by high-quality genome assemblies. With advancements in sequencing technology, an increasing number of Sciaenidae genomes have been sequenced in recent years. This includes key aquaculture species in China such as the large yellow croaker (*Larimichthys crocea*) [39], and small yellow croaker (*Larimichthys polyactis*) [40], *Collichthys lucidus* [41], *Miichthys miiuy* [42], *Sciaenops ocellatus* [43], *Nibea coibor* [44], *Nibea albiflora* [45], and *Argyrosomus regius* [46]. In this study, we firstly assembled a high-quality genome of *A. japonicus* using advanced sequencing technology. Compared to the previously published assemblies of *A. japonicus* [16,17], our assembly achieved a contig N50 of 30 Mb, an improvement over the previous values of 18.4 Mb [16] and 2.26 Mb [17], and consisting of only 181 contigs. We estimated that the genome size of *A. japonicus* is in the range of 702~755 Mb, and our assembly (708 Mb) aligns closely with the South African (SA) assembly (742 Mb), consistent with this range. However, comparisons between the CN and SA assemblies suggest that the South African and Chinese populations of *A. japonicus* might represent subspecies or even distinct species, as the two genomes exhibit significant divergence [17]. With the new assembly provided in this study, further analyses can now be conducted across all three genomes to assess whether the two CN genomes are more similar to each other, or if they show significant divergence from the SA genome, in terms of global sequence identity and shared gene clusters. This analysis could further inform the hypothesis regarding the potential for distinct species in China and South Africa. Additionally, haploid genome and the X and Y chromosome have been assembled with new technology in our study (Table S2). This significant advancement will aid in the development of sex markers and facilitate the detection of sex-determining genes in this species.

Research into fish sex-determining genes not only enhances our understanding of sex determination mechanisms but also has practical applications in sex control breeding. The sex of fish can typically be identified through the direct observation of external differences between males and females, as well as through anatomical examinations of the gonads and the histological analysis of gonadal tissues. However, sex-specific molecular markers provide a more effective method for sex identification. In this study, we utilized resequencing data from 38 individuals, combining correlation analysis with the assumption of the phenotypic and genotypic concordance for the XX-XY or ZZ-ZW sex determination systems. Our findings confirm that *A. japonicus* adopts an XX-XY sex determination system, resolving the previous speculation of a ZZ-ZW system proposed by Jackson et al. [11]. And we successfully mapped its sex determination region on chromosome 9. Furthermore, we found two indels larger than 10 bp in this region and designed primers for their verification, achieving an accuracy rate of 98%. Both molecular markers are located in the intron of *dmrt1*, suggesting a reasonable sex-determining candidate gene for *A. japonicus* and facilitating advancements in breeding control.

To date, an increasing number of fish sex-determining genes have been identified. For example, *dmy* is the sex-determining gene and is exclusively expressed in male Japanese *Oryzias latipes*, while *dmrt1* is closely associated with male half-smooth tongue sole. Other important genes include *hsd17b1*, *bcar1*, *amh*, *gsdf*, *cyp19*, and *pfpdz1*. Recent studies have indicated that the macroscopic karyotype of Sciaenidae is relatively stable, with only a few species exhibiting variations. Over 80% of the reported karyotypes for Sciaenidae species are characterized by the original 2n = 48t, such as *Sciaenops ocellatus* [47] and *Argyrosomus amoyensis* [48]. In our laboratory, we have conducted extensive research on the sex-determining genes of Sciaenidae species. In 2016, Cao et al. identified two genes—*dmrt1* and *sox3*—that were specifically expressed in testes in large yellow croaker. Conversely, the *foxl2* and *lcnanos3* genes were found to be expressed at higher levels in ovaries [49]. Lin et al. discovered that the *gsdf* and *amh* genes were exclusively expressed in testes after gonadal differentiation, suggesting that *dmrt1* is a key gene for male differentiation, as supported by RT-qPCR and reverse transcription techniques [50]. Our laboratory has also made strides in studying *Nibea coibor* and *Nibea albiflora*. Han et al. reported that the *Dmrt1*, *Dnah2*, and *Htr7* genes were specifically expressed in testes, while the *Trim36* gene was detected only in ovaries [51]. Additionally, transcriptome analysis by Sun et al. revealed that the expression levels of four genes—*Zn366*, *Dmrt1*, *Kank1*, and *Ats3*—were significantly higher in males than in females. Using qRT-PCR, they speculated that the *dmrt1* gene on chromosome 9 is a candidate for sex determination in *Nibea albiflora* [45]. Subsequently, our study fine-mapped the sex determination region in *Nibea albiflora*, finding only two genes, *dmrt1* and *dmrt3*. Thus, we hypothesize that *dmrt1* is the primary sex-determining gene in *Nibea albiflora*. For *Nibea coibor*, our study indicated that *fam114a2* and *gemin5* are highly expressed in males, suggesting their involvement in sex determination [44]. In *Collichthys lucidus*, a species with a complex sex determination system, studies have shown that *dmrt1* is exclusively expressed in testes and is a strong sex-determining candidate gene of this species. In the closely related species *Argyrosomus regius*, recent research has revealed unique duplications of *cfap157* and *dmrt3* near *dmrt1*, potentially implicating them in sex determination [46].

The *dmrt1* gene is highly conserved in the sex determination pathway [52]. Numerous studies have demonstrated that the *dmrt* gene is crucial for sexual gland development across various vertebrates, including half-smooth tongue sole [53], medaka fish [54], chicken [55], and African clawed frog [56]. It plays a role in mechanisms such as gene duplication [54], dosage effects [57], and epigenetic modifications that contribute to sex determination in different organisms. The homologous copy of *dmrt1*, known as *Dmrt1bY*, exists in the Y chromosome-specific region of *Oryzias latipes* and is vital for testis development. The deletion of *dmrt1* can result in the transformation of males into females, a phenomenon also observed in zebrafish. Our results showed that the *dmrt1* gene can be a possible candidate gene of sex determination in *A. japonicus,* but further molecular investigations are needed. Since there are sexual dimorphisms in maturation and growth, the sex markers that we developed will benefit sex-controlling aquaculture in *A. japonicus.*

## 5. Conclusions

In this study, we successfully assembled a high-quality chromosome-level genome of *A. japonicus*, with a size of 708.02 Mb. We utilized association analysis to identify the ninth chromosome as the sex chromosome. Furthermore, we developed two molecular markers within the sex determination region that effectively distinguish between male and female individuals. This result lays the groundwork for further research into sex determination and breeding control in *A. japonicus*, providing valuable tools for future studies.

## Figures and Tables

**Table 1:** Overview of estimated genome size of jellyfish.

K-mer Length	19	23	27	31
Total No. of fields	9386	9138	8834	8679
Peak	16	14	13	12
Total k-mers	11,246,682,729	10,458,199,060	9,733,006,649	9,067,661,088
Genome size	702,917,671	747,014,219	748,692,819	755,638,424
Single-copy	576,367,348	643,631,200	656,287,655	642,843,474
Proportion	0.820	0.862	0.877	0.851

**Table 2:** Statistics of contig and scaffold level genome assembly data.

Type	Contig (bp)	Scaffold (bp)
Number	181	109
N10	33,144,857	34,698,277
N50	30,007,843	30,008,023
N90	22,543,259	22,559,432
Max length	33,623,154	37,222,054
Total length	708,806,976	708,029,149

Note: N10, N50, and N90 are statistical measure of contig length, meaning that at least 10%, 50%, or 90% of the nucleotides in the assembly belongs to contigs with the N10, N50, or N90 length or longer, respectively.

**Table 3:** Statistics on the percentage of repetitive sequences in *A. japonicus*.

Repeat Element	Fragments	Total Length (bp)	% of Genome
LINE	78,183	19,481,765	2.75
SINE	29,084	4,236,640	0.60
LTR element	22,210	7,111,927	1.00
DNA element	147,730	22,987,630	3.43
RC element	5967	4,046,474	0.57
Simple_repeat	458,403	23,061,626	3.26
Small RNA	861	106,456	0.02
Satellite	2882	818,415	0.12
Other	109,790	15,331,373	2.16
Unclassified	416,610	87,700,519	12.38
Total	1,271,720	174,573,389	24.64

**Table 4:** Design table of two sex-specific molecular marker primers.

Primers	Primer Sequence (5′-3′)	Product Size (bp)
MFS-1	F: TATGTCTGGAGGTCACTG R: GCTTATTTGGAGGATTGT	364 bp/346 bp
MFS-2	F: TGTGAATGGGTGAATGAG R: TAGCTTTGTACTTTGTTCC	426 bp/412 bp

## Data Availability

All sequencing data were deposited in the National Center for Biotechnology Information (NCBI) SRA database under PRJNA1070049. The final chromosome assembly was deposited in GenBank at NCBI GCA_040802145.1.

## Supplementary Materials

The following supporting information can be downloaded at: https://www.mdpi.com/article/10.3390/genes15121493/s1, Figure S1. The CO I sequence-based phylogenetic tree was constructed using the Neighbor-joining method. Figure S2. Genetic distances between species based on CO I genes. Figure S3. Estimation of repetitive sequences and heterozygosity of *Argyrosomus japonicus* based on K-mer values of 19, 23, 27, and 31, respectively. Table S1. The top ten genes significantly associated with sex phenotypes. Table S2. Statistic of assembly of multiple Sciaenidae species. Red highlights the species provided in our study.
