# Supplementary material for: Genome Insights and Identification of Sex Determination Region and Sex Markers in Argyrosomus japonicus
Source: Genes (Basel). 2024 Nov 21;15(12):1493. doi: 10.3390/genes15121493 (PMC11675234; doi:10.3390/genes15121493)
Supplement: Supplementary file 1 [file genes-15-01493-s001.zip › genes-3280397-supplementary.pdf]

Genome Insights and Identification of Sex Determination Region and Sex markers in *Argyrosomus japonicus*

Yike Liu[1], Dinaer Yekefenhazi[1], Xianfeng Yang[2], Qihui Zhu[3], Kun Ye[1], Qiwei He[1], Fang Han[1*], Wanbo Li[1*], Dongdong Xu[3*]

[1] Key Laboratory of Healthy Mariculture for the East China Sea, Ministry of Agriculture and Rural Affairs, Jimei University, Xiamen 361021, China

[2]Agro-Tech Extension Center of Guangdong Province, Guangzhou, China

[3]Key Lab of Mariculture and enhancement of Zhejiang Province, Zhejiang Marine fisheries Research institute, 316100, Zhoushan, China

Figure S1 2

Figure S2 3

Figure S3 4

Table S1..5

Table S2..6

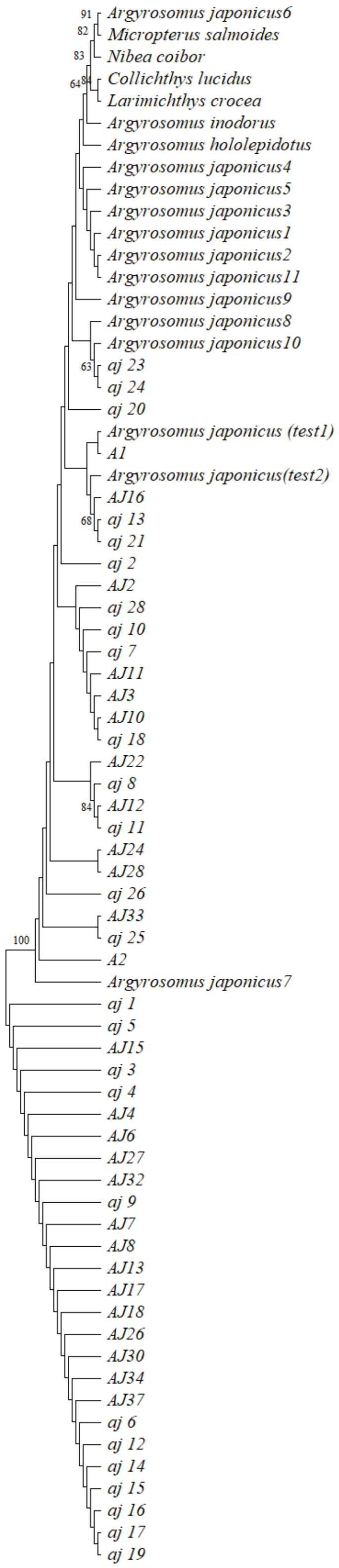

Figure S1. The CO I sequence-based phylogenetic tree was constructed using the Neighbor-joining method. (Note: AJ is the first batch of *Argyrosomus japonicus*, aj is the second batch of *Argyrosomus japonicus*) *Argyrosomus japonicus* 1-11 represent *Argyrosomus japonicus* downloaded from NCBI. Bootstrap values larger than 60 are indicated on the clade nodes.

Inter－species	1	2	3	4	5	6	7	8	9	10	11	12	13	14	15	16	17	18	19	20	21
1																					
2	0.002																				
3	0.002	0.003																			
4	0.002	0.004	0.000																		
5	0.003	0.005	0.002	0.000																	
6	0.218	0.222	0.218	0.228	0.216																
7	0.007	0.007	0.005	0.004	0.007	0.228															
8	0.006	0.008	0.005	0.004	0.006	0.215	0.000														
9	0.004	0.004	0.002	0.002	0.004	0.214	0.004	0.005													
10	0.006	0.008	0.005	0.004	0.006	0.216	0.000	0.000	0.004												
11	0.000	0.002	0.002	0.002	0.003	0.213	0.007	0.008	0.004	0.006											
12	0.012	0.014	0.010	0.006	0.012	0.225	0.000	0.005	0.007	0.005	0.012										
13	0.007	0.009	0.005	0.004	0.007	0.221	0.000	0.000	0.004	0.000	0.007	0.000									
14	0.022	0.023	0.020	0.006	0.018	0.231	0.000	0.015	0.018	0.015	0.022	0.003	0.000								
15	0.014	0.015	0.012	0.008	0.014	0.227	0.000	0.007	0.008	0.007	0.014	0.005	0.003	0.007							
16	0.025	0.028	0.023	0.018	0.025	0.218	0.027	0.028	0.029	0.028	0.024	0.035	0.029	0.045	0.036						
17	0.051	0.056	0.049	0.044	0.051	0.208	0.051	0.053	0.053	0.051	0.050	0.059	0.055	0.068	0.061	0.054					
18	0.169	0.171	0.170	0.172	0.169	0.229	0.165	0.167	0.166	0.167	0.169	0.171	0.166	0.174	0.172	0.182	0.157				
19	0.153	0.159	0.155	0.149	0.156	0.220	0.154	0.148	0.160	0.152	0.151	0.159	0.152	0.167	0.159	0.156	0.155	0.119			
20	0.164	0.169	0.162	0.166	0.161	0.207	0.168	0.161	0.162	0.162	0.160	0.172	0.168	0.179	0.174	0.160	0.148	0.172	0.183		
21	0.201	0.207	0.199	0.199	0.197	0.195	0.208	0.200	0.200	0.200	0.200	0.207	0.206	0.209	0.209	0.199	0.199	0.195	0.190	0.187	

Figure S2. Genetic distances between species based on CO I genes, species is as follows: *Argyrosomus japonicus* 1-11, *Argyrosomus japonicus* (test1), *Argyrosomus japonicus* (test2), A1, A2, *Argyrosomus hololepidotus*, *Argyrosomus inodorus*, *Collichthys lucidus*, *Larimichthys crocea*, *Nibea coibor*, *Micropterus salmoides*.

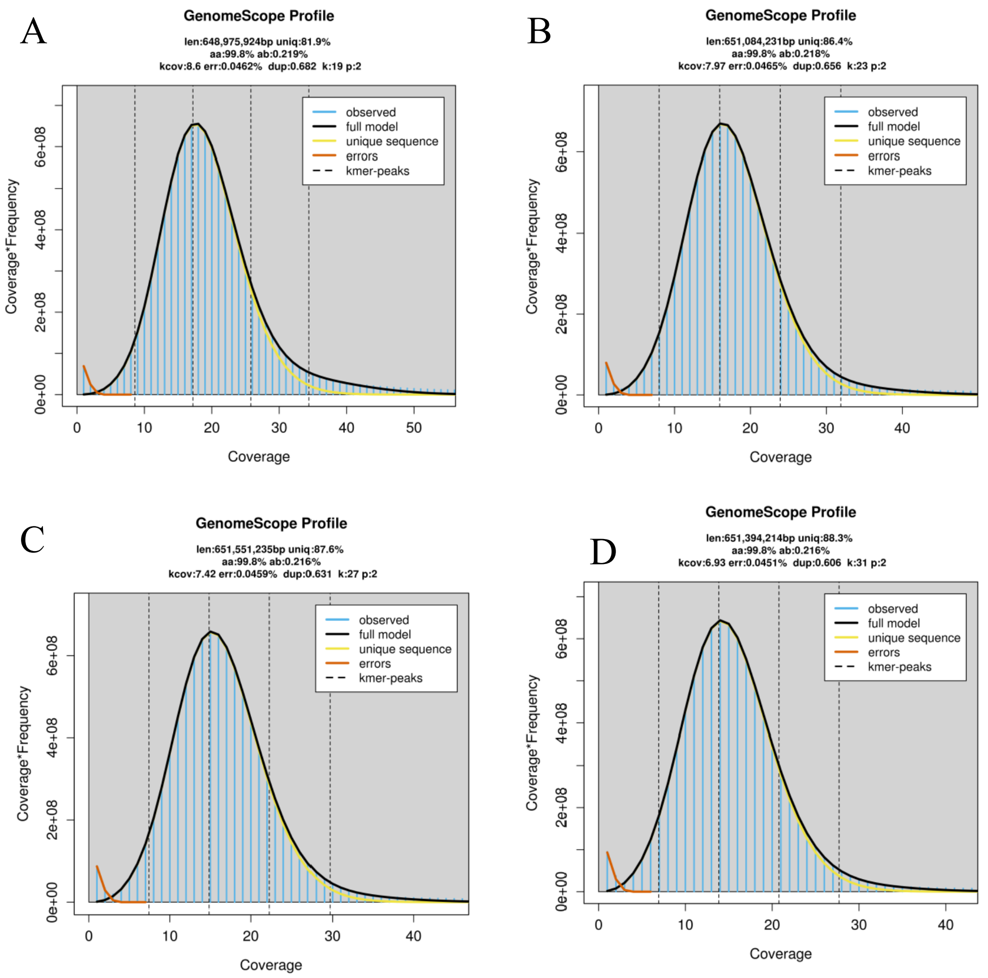

Figure S3. Estimation of repetitive sequences and heterozygosity of *Argyrosomus japonicus* based on K-mer values of 19, 23, 27, and 31, respectively.

Table S1. The top ten genes significantly associated with sex phenotypes.

Gene Name	Interval of Gene (CDS)	Physical position of most significant SNP	*P*-value
ptges2	Chr9:20711200-20714927	Chr9:20713771	8.35E-09
ttc16	Chr9:20275669-20282088	Chr9:20280815	1.36E-08
cfap299	Chr9:21447083 –21516276	Chr9:21478499	8.37E-08
wdfy3	Chr9:21753669- 21820040	Chr9:21763724	1.00E-07
dmrt1	Chr9:22571179- 22591499	Chr9:22589579	1.12E-07
zbed1	Chr9:22675107- 22676948	Chr9:20257929	1.22E-07
dapk1	Chr9:22366676-22400777	Chr9:22376725	1.22E-07
derl2	Chr9:20644557-20649937	Chr9:20647721	2.16E-07
snx2	Chr9:19929677-19952192	Chr9:19937067	3.07E-07
mapk	Chr9:22265754-22276774	Chr9:22268764	3.07E-07

Table S2. Statistic of assembly of multiple Sciaenidae species. Red highlights the species provided in our study.

**species**	Contigs	Congtig（Mb）	Contig N50 (Mb)	Chrome (Mb)	scaffold N50（Mb）	Chrome proportion (%)	Genes
Larimichthys crocea	1591	723.86	2.83	668.67	/	92.48	23,657
Larimichthys polyactis	/	/	1.21	706	4.52	/	25,233
Collichthys lucidus	2134	811.23	1.1	877	35.9	96.86	28,602
Miichthys miiuy	480	651.33	7.73	637.65	/	94.98	/
Sciaenops ocellatus	/	/	0.099	686.62	25.62	87.82	/
Nibea coibor	314	627.60	10.66	627,66	26.22	/	21,433
Nibea albiflora	/	/	/	589.3	22.37	98.17	
Argyrosomus regius	1012	696.25	7.81	696.27	27.87	92.85	24,589
Argyrosomus japonicus (P-CN)	1,984	791	13.1	665.4	29.4	/	23,730
Argyrosomus japonicus(SA)	1416	/	0.75	742	5.49	/	22,438
Argyrosomus japonicus(CN)	181	708.8	30	708.02	30	98	24,726

Note: P-CN ,Previous -Chinese; CN, this study of Chinese; SA, South African.
